# Benign and Malignant Tumors of the Hand: Patterns, Pathology, and Surgical Outcomes in a Large Retrospective Cohort

**DOI:** 10.3390/cancers17183079

**Published:** 2025-09-21

**Authors:** Fabiana Battaglia, Roberta Giuffrida, Marco Pagano, Luigi Troisi, Gabriele Delia

**Affiliations:** 1 Department of Plastic and Reconstructive Surgery, University Hospital of Messina “AOU Gaetano Martino”, 98125 Messina, Italy; fabiana.battaglia@studenti.unime.it (F.B.); gabriele.delia@unime.it (G.D.); 2 Department of Clinical and Experimental Medicine, Section of Dermatology, University of Messina, 98125 Messina, Italy; 3 Department of Orthopaedics, Udine Hospital, 33100 Udine, Italy; marco.pagano@policlinicoudine.it; 4 IRCCS MultiMedica Group, University Department of Hand Surgery and Rehabilitation, San Giuseppe Hospital, 20123 Milan, Italy; luigi.troisi@unimi.it

**Keywords:** hand neoplasms, benign hand tumors, malignant hand tumors, retrospective cohort, surgical outcomes, epidemiology

## Abstract

*Background:* Hand tumors encompass a heterogeneous spectrum of benign, malignant, and tumor-like lesions with diverse clinical behavior. While international studies have reported epidemiological and clinicopathological features, large-scale data in Italian populations are scarce. This retrospective analysis represents one of the largest Italian surgical series of histologically confirmed hand tumors. The objective was to evaluate clinicopathological characteristics, anatomical distribution, and surgical outcomes of these lesions over a 5-year period. *Methods:* A total of 250 patients who underwent surgery for hand tumors at the Department of Plastic and Reconstructive Surgery, University Hospital “G. Martino,” Messina, Italy, from January 2020 to December 2024, were retrospectively reviewed. Data from clinical records, imaging, and histopathology were categorized as tumor-like lesions, benign neoplasms, or malignant tumors. Demographic and clinical variables were compared across diagnostic groups. *Results:* The cohort included 127 males and 123 females (mean age 49.3 ± 18.6 years). Lesions were most frequently located in the digits (62%), followed by palm (21%), dorsum (11%), and wrist (6%). Tumor-like lesions represented 37.6% of cases, predominantly mucous cysts and foreign body granulomas. Benign tumors accounted for 49.2%, with giant cell tumors of the tendon sheath as the most common (31.7%). Malignant tumors were rare (10.4%), mainly squamous cell carcinoma, basal cell carcinoma, and melanoma. Patients with malignant lesions were significantly older (67.4 years) compared with those with benign or tumor-like lesions (*p* < 0.01). *Conclusions:* Benign and tumor-like lesions predominate among hand tumors, whereas malignancies are infrequent but clinically important. Surgical excision remains the treatment of choice, guided by preoperative imaging and confirmed histopathologically. Expanding this cohort and integrating molecular diagnostics with patient-reported outcomes may enhance future management strategies.

## Simple Summary

Hand tumors, although relatively rare, can have a significant impact on function, appearance, and quality of life. Most of these lesions are benign or tumor-like, but some can be malignant and require timely diagnosis and treatment. In this study, we reviewed 250 cases of surgically treated hand tumors over a five-year period in one of the largest Italian series to date. We analyzed the types of tumors, their frequency, where they were located, and how patients were treated. Our results confirm that benign lesions are by far the most common, while malignant tumors are rare but important to detect early. Understanding these patterns can help doctors recognize hand tumors sooner, choose the best treatment, and preserve both the function and the appearance of the hand. This knowledge can also guide future research to improve patient care and outcomes.

## 1. Introduction

Hand tumors, although relatively rare, are clinically significant because even small lesions can impair fine motor function, cause pain, or compromise appearance. Their constant visibility also carries a psychological and social burden, making accurate diagnosis and timely treatment essential. Hand tumors represent a heterogeneous spectrum of lesions, ranging from benign neoplasms and tumor-like conditions to rare malignancies. Although they account for a relatively small proportion of total neoplasms, their location in the hand has profound implications for function, esthetics, and patient quality of life. Soft-tissue tumors of the hand are relatively uncommon compared to other body sites, and the vast majority are benign, with malignancies representing only a small minority [1,2,3].

Among benign entities, ganglion cysts are the most common, accounting for approximately 65% of all soft tissue hand masses; they occur more frequently in women (about 3:1) and typically affect individuals aged 20–40 years [1]. Localized tenosynovial giant cell tumors (or giant cell tumors of the tendon sheath) follow as the second most frequent tumor type, with a recurrence rate of ~8.5% [4].

The epidemiology of hand bone tumors has been detailed in multiple retrospectives. In one series of 155 benign bone tumors (1992–2015), the mean patient age was 39.9 years, with most lesions located in the digits and managed primarily by intralesional curettage; pathological fractures occurred in approximately half of these patients [5]. Another large analysis of 631 primary skeletal tumors of the hand reported that most lesions were cartilage-derived, with bone cysts and osteogenic tumors being less common. The majority involved the phalanges, and only about 10% were malignant [6].

Comprehensive institutional reviews have further underscored the rarity of malignancy in hand tumors. For instance, a French retrospective study of 623 cases confirmed the predominance of benign soft tissue tumors, while malignancies were exceedingly rare. Similarly, a surgical series of 402 cases corroborated both the rarity of malignancy and the variation in distribution by site and age [7].

Beyond these common entities, rare and tumor-like lesions such as aneurysmal bone cysts [8], primary lymphoma of the nerves [9,10], or myoepithelioma remain underdocumented, despite their clinical relevance and potential for misdiagnosis.

Tumor-like lesions and rare benign tumors, such as glomus tumors, schwannomas, neurofibromas, epidermal inclusion cysts, and idiopathic tenosynovitis, are often under-documented despite being clinically relevant. A retrospective series of 80 patients treated between 2014 and 2020 described the clinical, imaging, and histopathologic features of such lesions and emphasized the need for specialist knowledge to avoid diagnostic and therapeutic errors [11].

Although studies provide valuable insights into tumor distribution, histology, and demographic profiles, the majority remain single-center and lack epidemiological data from large Italian cohorts. 

This gap is particularly relevant in Mediterranean countries, where large datasets are scarce. The present retrospective study of 250 surgically treated hand tumors represents one of the largest Italian series. By systematically analyzing patient demographics, clinical presentation, anatomical localization, and histopathologic spectrum, and contextualizing these findings with prior international evidence, this study aims to provide novel epidemiological insights and support evidence-based clinical management. 

## 2. Materials and Methods

This retrospective study included patients treated at the Department of Plastic and Reconstructive Surgery of the University Hospital ‘G. Martino,’ Messina, Italy, in collaboration with the Department of Pathological Anatomy. Institutional ethics committee approval was obtained prior to data collection, which encompassed all patients surgically managed between January 2020 and December 2024.

All medical records, surgical registries, and histopathological reports were reviewed. The following information was documented for each patient: age, sex, year of diagnosis, clinical presentation, anatomical location of the lesion, histopathological features including tumor type and dignity (tumor-like, benign, or malignant), and final diagnosis.

All cases included were surgically treated. Tumor specimens were excised and processed using standard histopathological techniques: paraffin embedding following EDTA decalcification for osseous lesions, sectioning into fresh slides, and staining with hematoxylin and eosin. Immunohistochemical analysis was performed when necessary to refine diagnostic classification. All specimens were reviewed by experienced pathologists specializing in musculoskeletal tumors. Histopathological evaluation was performed by experienced pathologists with full access to clinical and radiological information.

Radiological investigations, including plain radiographs, ultrasound, and magnetic resonance imaging (MRI), were performed preoperatively for most cases to assess tumor size, depth, and possible involvement of adjacent structures. For suspected malignant or metastatic tumors, a comprehensive oncological staging was performed, including laboratory studies and computed tomography of the thorax, abdomen, and pelvis.

Exclusion criteria were metastatic tumors to the hand, purely inflammatory or infectious lesions without neoplastic characteristics, and incomplete clinical or histological data.

Lesions were classified into three major categories:

Tumor-like lesions, such as mucous cysts, foreign body granulomas, pyogenic granulomas, epidermoid cysts, viral warts, and neuromas;

Benign neoplasms, including giant cell tumor of tendon sheath, hemangiomas, lipomas, glomus tumors, fibromas, keratoacanthomas, osteochondromas, schwannomas, neurofibromas, angioleiomyomas, myopericytomas, benign fibrous histiocytomas, enchondromas, osteoid osteomas, osteoblastomas, chondroblastomas, and lymphangiomas;

Malignant tumors, including squamous cell carcinoma, basal cell carcinoma, melanoma, epithelioid hemangioendothelioma, liposarcoma, and combined spinobasocellular carcinoma.

Data were entered into a structured database and statistically analyzed using IBM SPSS Statistics, version 29.0 (IBM Corp., Armonk, NY, USA). Continuous variables were expressed as mean ± standard deviation or median (range), and categorical variables as frequencies and percentages. Comparative analyses between benign and malignant lesions were performed using Chi-square or Fisher’s exact test for categorical variables and Student’s *t*-test or Mann–Whitney U test for continuous variables. Statistical significance was set at a two-tailed *p* < 0.05.

## 3. Results

**Figure 1:**
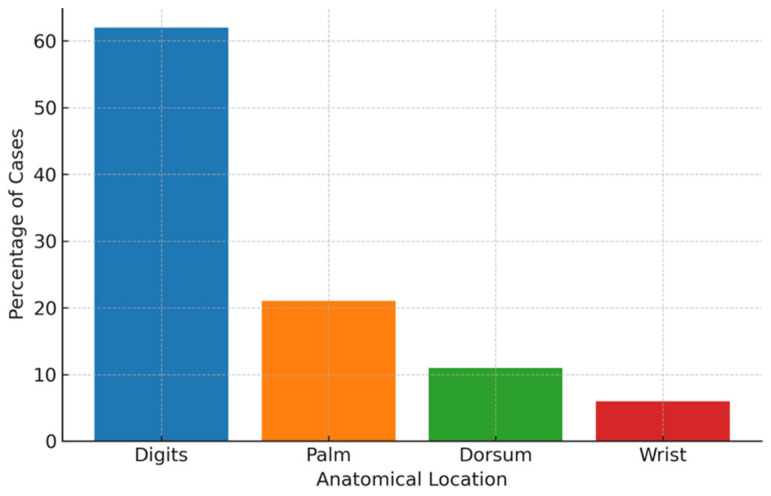
Distribution of hand tumors by anatomical location.

A total of 250 patients (127 males, 123 females) underwent surgical excision of hand tumors or tumor-like lesions between 2020 and 2024. The mean age at diagnosis was 49.3 ± 18.6 years (range: 1–92). Lesions were located most frequently in the digits (62%), followed by the palm (21%), dorsum of the hand (11%), and wrist (6%) as illustrated in Figure 1. 

Tumor-like lesions accounted for the majority of cases (37.6%), benign neoplasms comprised 49.2%, and malignant tumors represented 4.0% of all lesions.

### 3.1. Tumor-like Lesions

Among the 94 tumor-like lesions identified, the most common diagnoses were mucous cysts (*n* = 24), foreign body granulomas (*n* = 24), pyogenic granulomas (*n* = 18), epidermoid cysts (*n* = 11), viral warts (*n* = 10), and neuromas (*n* = 5). Tumor-like lesions were more common in females (56%) than in males (44%), with a median age of 48 years (range: 19–85). The most frequently involved sites were the distal phalanges and dorsal aspect of the digits as shown in Table 1. 

### 3.2. Benign Neoplasms

A total of 123 benign tumors were surgically excised during the study period. The most common benign neoplasms were giant cell tumors of the tendon sheath (*n* = 39), hemangiomas (*n* = 15), lipomas (*n* = 13), glomus tumors (*n* = 12), and fibromas (*n* = 11).

Less frequent entities included keratoacanthomas (*n* = 11), osteochondromas (*n* = 7), schwannomas (*n* = 5), neurofibromas (*n* = 4), and other rare benign tumors. Benign lesions were more prevalent in females (56%) and predominantly affected patients in their 4th and 5th decades of life as detailed in Table 2.

### 3.3. Malignant Tumors

Malignant tumors were rare, accounting for 26 cases (10.4%). The most frequent malignant histologies were squamous cell carcinoma (*n* = 10), basal cell carcinoma (*n* = 8), and melanoma (*n* = 5). Other rare malignant entities included epithelioid hemangioendothelioma (*n* = 1), liposarcoma (*n* = 1), and combined spino-basocellular carcinoma (*n* = 1). Malignant lesions were slightly more common in males (56%) and typically occurred in older patients (mean age 67.4 years, range: 45–90) as summarized in Table 3.

### 3.4. Statistical Analysis

Benign and tumor-like lesions were significantly more common than malignant tumors (*p* < 0.001). Malignant tumors were associated with older age compared to benign and tumor-like lesions (mean 67.4 vs. 49.3 years, *p* < 0.01). No significant sex difference was observed across the three diagnostic categories (Chi-square, *p* = 0.12).

## 4. Discussion

Lesions of the hand, including cystic formations, tumefactive masses, and neoplasms, are commonly encountered in clinical practice owing to the visibility and functionality of the hand. The immediate impact on esthetics and daily activities often prompts early medical consultation. However, as substantiated by our cohort and existing literature, most of these lesions are benign or reactive in nature, necessitating clinician vigilance but often resulting in simple reassurance [1].

Hand tumors remain relatively uncommon, and most are benign. Our study provides additional large-scale data from Italy, helping to fill a gap in Mediterranean populations where few series have been reported [12].

Population variations were further underlined by a multivariate study from Singapore, which found that exposure to the environment and ethnic background had an impact on incidence and presentation [13].

Large multicenter studies in Europe and North America confirm the predominance of benign lesions and the rarity of malignancies [14].

A recent multicenter review of over 4000 upper extremity tumors reported similar distributions, confirming the predominance of benign lesions across different geographic regions and healthcare systems [15].

These results are consistent with multiple large series indicating that benign or tumor-like lesions account for 90–98% of hand tumors, whereas malignancy occurs in only ~1–5% of cases [2,7,16].

Our Italian series adds further evidence from a Mediterranean population, where large cohorts remain scarce. A comparative study from Singapore underscores variability tied to demographic factors: malignancy rates remain low, with darker skin pigmentation correlated with reduced ultraviolet-induced malignancy risk, a meaningful insight into population-specific differences [13].

Non-neoplastic masses, such as mucous cysts, foreign-body granulomas, and pyogenic granulomas, comprised nearly 38% of our cohort, consistent with other institutional reports. Particularly, mucous cysts and ganglions are frequently related to repetitive microtrauma and joint overuse [1]. Among benign tumors, tenosynovial giant cell tumor (GCTTS) emerged as the most prevalent entity, accounting for ~36–40% of benign soft tissue tumors in both our series and published studies.

Despite its benign classification, GCTTS poses relevant clinical challenges due to local aggressiveness and a recurrence rate ranging from 5% to 15%. Surgical excision with meticulous dissection under magnification has been shown to significantly reduce recurrence rates compared with simple excision. Furthermore, the Al-Qattan classification differentiates encapsulated (type I) from infiltrative (type II) tumors, the latter associated with higher recurrence [17].

The recent literature underscores the neoplastic origin of GCTTS, largely associated with CSF1 gene fusions, its risk of local recurrence, and the emerging role of targeted therapies such as CSF1R inhibitors, particularly in diffuse forms [4,17].

Although usually benign, their clinical overlap with neoplasms emphasizes the importance of histological confirmation. Modern imaging techniques, including high-resolution ultrasound and diffusion-weighted MRI, have improved diagnostic accuracy and surgical planning, reducing unnecessary excisions. 

Anatomically, over two-thirds of our cases were located in the digits (62%), followed by palm (21%), dorsum (11%) and wrist (6%). This distribution mirrors Simon et al.’s famous registry data: 70% involvement of phalanges, followed by metacarpals and carpals, with malignancy most likely in carpal lesions (8.6%) and metacarpal (12.2%) [6].

Benign bone tumors of the hand, although relatively uncommon, warrant attention due to their potential for pathological fractures and functional impairment. Enchondromas, osteochondromas, and other cartilage-derived tumors represent the majority of these lesions [6,18]. Recent studies have demonstrated favorable outcomes with simple curettage followed by impaction grafting using allogeneic bone, showing low recurrence rates and excellent preservation of function [19].

However, predictors of recurrence and complications include tumor size greater than 5 cm and location within phalanges, which should guide both surgical planning and patient counseling [20].

Primary malignancies of the hand—predominantly skin tumors (SCC, BCC, melanoma)—represented ~90% of malignant presentations in prior studies [1,2]. Our data corroborate this, with squamous cell carcinoma being the most frequent. Rare mesenchymal sarcomas (e.g., epithelioid hemangioendothelioma or glomangiosarcoma) are distinctly uncommon but clinically significant when present.

Epithelioid sarcoma merits special attention: although extremely rare, it often arises in the distal extremities, such as the hand, and is frequently mistaken for a benign lesion, leading to delayed diagnosis and treatment [21].

In fact, some series report that up to 13% of patients present with multifocal or metastatic disease at the time of diagnosis, underlining its aggressive potential [22].

On the therapeutic front, there has been important progress: in a pivotal phase II trial, the EZH2 inhibitor tazemetostat demonstrated clinical activity in patients with advanced or metastatic epithelioid sarcoma, showing an objective response rate of around 15% and a manageable safety profile [23].

Cutaneous metastases to the hand are exceedingly rare, with incidence estimates between 0.6% and 10% in oncology patients, depending on the primary tumor type [24]. Acral metastases, metastases to fingers or hand bones, account for just 0.0016–0.5% of all metastatic skin lesions [25]. The systemic review by Umana et al. (2021) reported lung cancer as the most frequent primary source (36.8%), followed by gastrointestinal and urinary malignancies [26].

Because benign lesions are frequent, there is a risk of under-treatment or diagnostic delay. However, increasing medico-legal scrutiny requires careful evaluation. Detailed history, targeted examination, and imaging (ultrasound or MRI) are essential, particularly in rapidly growing, painful, or suspicious lesions [1,27].

Recent studies have shown that advanced imaging can improve diagnostic accuracy and guide surgical planning [28].

Unplanned excisional biopsies, especially of suspected sarcomas, can lead to compromised outcomes and legal consequences. Multidisciplinary evaluation and referral to specialist hand surgery or orthopedic oncology centers remain best practice [29].

Finally, limb-sparing surgery and modern reconstructive techniques have brought remarkable progress in treating malignant hand tumors. Whenever possible, surgeons now favor preserving digits or hand function through meticulous tumor removal and reconstruction with local or regional flaps rather than opting for amputation. This approach achieves not only better oncological safety but also superior functional recovery and preservation of quality of life. A recent large case series including forearm and hand sarcomas demonstrated favorable long-term outcomes with limb-sparing resection and reconstruction, supporting its viability and effectiveness in experienced hands [30].

In this regard, our study contributes valuable data from an Italian center, helping to fill an existing gap in Mediterranean populations where large-scale cohorts remain scarce. The integration of multidisciplinary expertise, including plastic surgeons, orthopedic oncologists, radiologists, and pathologists, represents a cornerstone in optimizing patient outcomes. Such collaboration not only improves diagnostic accuracy and therapeutic decision-making but also ensures that functional and psychosocial aspects are prioritized alongside oncological safety, thereby promoting holistic, patient-centered care.

Recent literature highlights under-recognized lesions such as myoepithelioma of the hand, frequently misdiagnosed as lipomas or fibromas, requiring nuanced histology and immunohistochemistry for accurate identification [31]. Albanese et al. (2024) also summarized a spectrum of rare benign bone tumors (bizarre parosteal osteochondromatous proliferation, solid aneurysmal bone cyst, epidermoid cyst), proposing expanded diagnostic consideration beyond classic enchondromas [18].

Our study’s retrospective single-center design and current sample size of 250 cases may limit generalizability. Future prospective studies incorporating larger cohorts and standardized functional outcome assessments are warranted to strengthen statistical power and enhance the understanding of hand tumor epidemiology. Additionally, molecular characterization (e.g., CSF1 fusions in GCTTS or fusion-gene driven tumors) may further differentiate benign from borderline lesions and guide precision therapies [17].

This study has several limitations. First, its retrospective and single-center design may introduce referral bias, as our institution is a tertiary referral hospital. Second, the sample size, although one of the largest Italian series, may still limit the generalizability of the findings. Third, long-term functional outcomes and patient-reported measures were not available, precluding evaluation of functional sequelae after surgery. Finally, despite all specimens being reviewed by experienced pathologists, potential diagnostic or interpretative bias in histopathology cannot be completely excluded.

## 5. Conclusions

This retrospective study, encompassing one of the largest Italian series of histologically confirmed hand tumors, reinforces the predominance of benign and tumor-like lesions while underscoring the rarity of malignancies. Our findings, consistent with the international literature, highlight that most hand lesions can be effectively managed through surgical excision, with malignancies representing only a small fraction of cases. Nevertheless, their functional and esthetic implications warrant meticulous clinical evaluation, supported by modern imaging and histopathological analysis. The integration of molecular diagnostics and multidisciplinary decision-making may further refine diagnostic accuracy and therapeutic strategies, particularly for rare or borderline lesions. Incorporating prospective functional outcome assessments in future studies will strengthen epidemiological understanding and contribute to evidence-based, patient-centered management of hand tumors.

In comparison with previous European and international series, our Italian cohort confirms the predominance of benign lesions but shows a slightly higher proportion of malignant tumors (10.4%). This may reflect referral bias or population-specific factors in a Mediterranean setting, where large datasets are scarce. These findings provide novel epidemiological insights and complement existing international evidence.

## Figures and Tables

**Table 1:** Distribution of tumor-like lesions of the hand.

Lesion Type	Total (*n*)	Male (*n*)	Female (*n*)	Age Range (yrs)
Mucous cyst	24	6	18	19–84
Foreign body granuloma	24	13	11	19–73
Pyogenic granuloma	18	7	11	23–85
Epidermoid cyst	11	10	1	32–61
Viral wart	10	8	2	24–76
Neuroma	5	5	3	22–49
Total	94	49	46	

**Table 2:** Benign tumors of the hand.

Tumor Type	Total (*n*)	Male (*n*)	Female (*n*)	Age Range (yrs)
Giant cell tumor of the tendon sheath (GCTTS)	39	14	26	14–81
Hemangioma	15	6	9	25–58
Lipoma	13	6	7	24–81
Glomus tumor	12	4	8	23–62
Fibroma	11	8	3	20–65
Keratoacanthoma	11	2	9	70–92
Osteochondroma	7	2	5	23–71
Schwannoma	5	3	2	73–75
Neurofibroma	4	1	3	32–70
Other benign tumors *	6	5	1	16–45
Total	123	51	72	

* Includes angioleiomyoma, myopericytoma, benign fibrous histiocytoma, enchondroma, osteoid osteoma, osteoblastoma, chondroblastoma, lymphangioma.

**Table 3:** Malignant tumors of the hand.

Tumor Type	Total (*n*)	Male (*n*)	Female (*n*)	Age Range (yrs)
Squamous cell carcinoma	10	7	3	47–87
Basal cell carcinoma	8	7	1	51–80
Melanoma	5	3	2	45–90
Epithelioid hemangioendothelioma	1	0	1	33
Liposarcoma	1	1	0	46
Spino-basocellular carcinoma	1	1	0	84
Total	26	19	7	

## Data Availability

The data presented in this study are available on reasonable request from the corresponding author. The data are not publicly available due to privacy and ethical restrictions.

## Abbreviations

The following abbreviations are used in this manuscript:

BCC	Basal cell carcinoma
CT	Computed tomography
CSF1R	Colony-Stimulating Factor 1 Receptor
EDTA	Ethylenediaminetetraacetic Acid
GCTTS	Giant Cell Tumor of the Tendon Sheath
H&E	Hematoxylin and Eosin
MRI	Magnetic Resonance Imaging
SCC	Squamous Cell Carcinoma
